# Ganglioneuroblastoma u Dziecka z Przewlekłym bólem Brzucha ‒ Opis Przypadku

**DOI:** 10.34763/devperiodmed.20182204.364370

**Published:** 2019-01-14

**Authors:** Tatiana Jamer, Tomasz Pytrus, Urszula Zaleska-Dorobisz, Barbara Iwańczak

**Affiliations:** 1 II Katedra i Klinika Pediatrii, Gastroenterologii i Żywienia, Uniwersytet Medyczny im. Piastów Śląskich we Wrocławiu, Wrocławi Polska; 2 Zakład Radiologii Ogólnej i Pediatrycznej, Uniwersytet Medyczny im. Piastów Śląskich we Wrocławiu, Wrocławi Polska

**Keywords:** bóle brzucha, ganglioneuroblastoma, dzieci, abdominal pain, ganglioneuroblastoma, children

## Abstract

Przewlekłe bóle brzucha są bardzo często występującą dolegliwością w populacji dzieci i młodzieży. W większości przypadków podłożem są czynnościowe zaburzenia przewodu pokarmowego. Jednak u kilku procent dzieci powodem utrzymującego się przewlekle bólu brzucha są schorzenia organiczne zlokalizowane w obrębie przewodu pokarmowego i poza przewodem pokarmowym, w tym również procesy nowotworowe. Wśród przyczyn organicznych, oprócz tych często spotykanych, takich jak: nietolerancje i alergie pokarmowe, choroba refluksowa przełyku, przewlekle zapalenie żołądka i dwunastnicy czy zakażenia układu moczowego, w diagnostyce należy uwzględnić również przyczyny bardzo rzadkie, na przykład choroby nowotworowe, a wśród nich guzy w obrębie jamy brzusznej. W opisywanym przez nas przypadku, u 6-letniej dziewczynki z przewlekłymi bólami brzucha, objawami o charakterze refluksu żołądkowo-przełykowego i zaparciami, z rozpoznaną wcześniej alergią pokarmową i nietolerancją laktozy, z uwagi na wystąpienie objawów alarmowych, ustalono wskazania do poszerzenia diagnostyki. Uwidoczniony w badaniu radiologicznym klatki piersiowej cień krągły na wysokości przepony, okazał się być w badaniu komputerowym, guzem okolicy przykręgosłupowej z cechami charakterystycznymi dla neuroblastoma. Po makroskopowo całkowitej resekcji guza, na podstawie wyniku badania histopatologicznego, ustalono rozpoznanie: ganglineuroblastoma. Obecność objawów alarmowych w badaniu podmiotowym i przedmiotowym u dzieci z bólami brzucha sugeruje wyższe prawdopodobieństwo organicznej przyczyny dolegliwości i zawsze powinno skłaniać do przeprowadzenia poszerzonej diagnostyki. Nerwiak zarodkowy zwojowy (ganglioneuroblastoma) należy do chorób bardzo rzadkich, w większości przypadków zlokalizowany jest pierwotnie w obrębie jamy brzusznej, a najczęstszym związanym z nim objawem jest ból brzucha.

## Abstract (en)

Chronic abdominal pain is a very common complaint in the population of children and adolescents. In most cases, the usual cause are functional gastrointestinal disorders. However, in a few percent of children, the reason for persistent chronic stomach pain are organic diseases occurring in the gastrointestinal tract, as well as parenteral diseases, including uro-genital tract abnormalities, inflammation of the lower respiratory tract and cancer processes. Among organic causes, in addition to those commonly encountered, such as: intolerances and food allergies, gastroesophageal reflux disease, chronic gastritis or duodenitis, or urinary tract infections, the diagnosis should also include very rare causes, for example, neoplastic diseases, among them tumors of the abdominal cavity. In the case described in the present article, a 6-year-old girl with chronic abdominal pain, symptoms of gastro-oesophageal reflux and constipation, and previously diagnosed food allergy and lactose intolerance, was referred for widening the diagnosics due to the occurrence of alarm symptoms. The nodule revealed in the chest X-ray, in CT scan, turned out to be a paravertebral tumor with the specific features of neuroblastoma. After a macroscopically complete tumor resection based on the result of histopathological examination, the diagnosis of ganglineuroblastoma was established. The presence of alarm symptoms in anamnesis and physical examination in children with abdominal pain suggests a higher probability of the organic origin of the disease and should always lead to extended diagnostics. Ganglioneuroblastoma is a very rare disease, in most cases is located primarily in the abdominal cavity, and the most common associated symptom is abdominal pain.

## Wprowadzenie

Przewlekły ból brzucha, rozumiany jako długo utrzymujący się, ciągły bądź nawracający, dotyczy znacznej części populacji dzieci i młodzieży. Częstość występowania przewlekłych bólów brzucha u dzieci nie jest dokładnie poznana. Szacuje się, że problem ten jest powodem od 2 do 4% wszystkich wizyt w gabinetach pediatrycznych [1]. W badaniu Hyams i wsp. [2] dotyczącym częstości występowania objawów gastroenterologicznych wśród młodzieży, bóle brzucha notowano aż u 75% wszystkich badanych, a dolegliwości bólowe przynajmniej jeden raz w tygodniu zgłaszało 13% uczniów szkół gimnazjalnych i 17% uczniów szkół licealnych. U większości dzieci dolegliwość ta ma charakter czynnościowy, bez obiektywnie stwierdzanej przyczyny organicznej. Jednak u około 3-8% powodem przewlekłego bólu brzucha są choroby organiczne występujące w obrębie przewodu pokarmowego, jak również schorzenia pozajelitowe, takie jak nieprawidłowości w obrębie dróg moczowo-płciowych, stany zapalne dolnych dróg oddechowych czy procesy nowotworowe [3]. Nowotwory w wieku rozwojowym są chorobami bardzo rzadkimi. Obecnie w Polsce notuje się około 1000 zachorowań rocznie [4, 5]. U dzieci najczęściej występują białaczki, które stanowią około 26% zachorowań. Drugie miejsce zajmują guzy centralnego układu nerwowego. Trzecią co do częstości przyczyną zmian nowotworowych u dzieci (około 7% wszystkich nowotworów diagnozowanych u dzieci) są guzy wywodzące się z pierwotnych komórek współczulnego układu nerwowego, do których zaliczane są nerwiak zarodkowy (neuroblastoma, NB) oraz wyżej zróżnicowany nerwiak zarodkowy zwojowy (ganglioneuroblastoma, GNB) [6, 7]. Manifestacja kliniczna obu guzów jest podobna, uwarunkowana lokalizacją i zaawansowaniem choroby, obecnością przerzutów oraz aktywnością wydzielniczą guza. W większości przypadków guz zlokalizowany jest pierwotnie w obrębie jamy brzusznej (nadnercza, przestrzeń zaotrzewnowa), a najczęstszej zgłaszanym objawem są bóle brzucha (53% pacjentów z NB i 68% z GNB) [6].

## Opis przypadku

Niespełna 6-letnia dziewczynka została przyjęta do kliniki z powodu przewlekłych bólów brzucha, które uległy nasileniu w ciągu ostatnich kilku dni przed przyjęciem. We wstępnej ocenie, uwagę zwracała również tendencja do zaparć utrzymująca się od okresu niemowlęcego oraz objawy refluksu żołądkowo-przełykowego. Wywiad rodzinny był nieobciążony. Dziewczynka urodziła się z ciąży pierwszej, porodu o czasie, drogą cięcia cesarskiego z powodu położenia miednicowego płodu, z prawidłową masą ciała, w stanie dobrym (9 punktów Apgar). Karmiona była piersią do 2 m.ż., następnie z powodu nasilonych kolek, wprowadzona została mieszanka o znacznym stopniu hydrolizy, z dobrym efektem. Dieta była rozszerzana, zgodnie z zaleceniami, od 6 m.ż. Od tego czasu występowały zaparcia. Dziewczynka oddawała stolce co kilka dni z wysiłkiem, o twardej konsystencji, bez patologicznych domieszek, przyjmowała postawę retencyjną. Od około 4 r.ż. zaczęła zgłaszać bóle brzucha. Po 3 miesiącach utrzymywania się dolegliwości, została skierowana do oddziału pediatrycznego szpitala rejonowego celem diagnostyki. Wykonane wówczas badania laboratoryjne, poza nieznaczną nadpłytkowością, pozostawały w granicach normy, w tym badanie serologiczne w kierunku choroby trzewnej (przeciwciała przeciw transglutaminazie tkankowej w klasach IgA i IgG). W badaniu ultrasonograficznym jamy brzusznej opisany został prawidłowy obraz narządów wewnętrznych i przestrzeni zaotrzewnowej. Po kilku dniach pobytu w domu, z powodu nasilenia dolegliwości bólowych brzucha z podejrzeniem niedrożności jelit, przyjęta została ponownie do szpitala. Po wykluczeniu potrzeby interwencji chirurgicznej (na podstawie prawidłowego wyniku badania USG jamy brzusznej i obserwacji klinicznej), dziewczynka została wypisana do domu z zaleceniami stosowania preparatu makrogolu i wlewek doodbytniczych. Leczenie było kontynuowane w domu z przejściową poprawą. Po kolejnych czterech miesiącach, z powodu nawracających bólów brzucha i zaparć, ponownie hospitalizowana była w oddziale pediatrycznym. W toku przeprowadzonej diagnostyki, u pacjentki zostały ustalone rozpoznania: nietolerancja laktozy, alergia pokarmowa oraz zaparcia. W leczeniu zastosowana została dieta eliminacyjna, która od tego czasu była ściśle przestrzegana. Po 3 miesiącach ponowne nasiliły się dolegliwości bólowe zlokalizowane w nadbrzuszu oraz dodatkowo dziewczynka uskarżała się na bóle o charakterze kłucia w okolicy lewego podżebrza, wybudzające kilkakrotnie w nocy. Ponadto występowały nasilone odbijania, nawracające czkawki, uczucie cofania pokarmu, poranna chrypka, chrząkanie, uczucie ciała obcego w gardle, od dłuższego czasu pokasływanie. Stolce oddawała co 3 dni, z wysiłkiem, o zbitej konsystencji, bez patologicznych domieszek. Dziewczynka wykazywała niezaburzoną aktywność fizyczną oraz prawidłowe łaknienie. W wywiadzie zwracał uwagę ubytek masy ciała około 1 kg w ciągu ostatnich 4 tygodni.

Przy przyjęciu do kliniki dziewczynka była w stanie ogólnym dobrym, eutroficzna (masa ciała 50-75 centyl, wzrost 50 centyl, BMI 50-85 centyl). W badaniu fizykalnym, poza niewielką skoliozą w odcinku piersiowym i ubytkami próchniczymi, nie stwierdzono odchyleń od normy. Wykonane badania laboratoryjne z nieprawidłowości ujawniły jedynie nieznacznie przyśpieszone OB (16 mm), przy prawidłowym CRP (0,4 mg/l) oraz liczbę płytek krwi powyżej górnej granicy normy (PLT 504 10^3/uL). W badaniu ultrasonograficznym jamy brzusznej został opisywany prawidłowy obraz narządów wewnętrznych i przestrzeni zaotrzewnowej.

W trakcie pobytu w klinice dziewczynka codziennie zgłaszała ból o charakterze kłucia zlokalizowany w lewym podżebrzu, również wybudzający ze snu. Ponadto utrzymywał się suchy kaszel o niewielkim nasileniu. W wykonanym zdjęciu RTG klatki piersiowej uwidoczniony został w rzucie kręgosłupa, na wysokości przepony, słabo odgraniczony krągły cień wielkości około 4x4cm, mogący sugerować przepuklinę przeponową. Zdjęcie RTG klatki piersiowej z widoczną zmianą przedstawiono na rycinie 1.

Wynik wykonanego badania kontrastowego górnego odcinka przewodu pokarmowego nie potwierdził obecności przepukliny przeponowej. Dziewczynka została zakwalifikowana do badania TK klatki piersiowej z kontrastem, które uwidoczniło przykręgosłupowo po stronie prawej (na wysokości Th9-L2) hipodensyjną, miękkotkankową masę o wymiarach 1,4x3,5x5,1 cm, z drobnymi zwapnieniami, przylegającą do trzonów kręgów i tylnych odcinków dolnych prawych żeber, do opłucnej i do prawej odnogi przepony, ponadto obniżenie wysokości trzonów kręgów Th7 i 8 opisane jako stan po złamaniu kompresyjnym oraz słabo różnicujące się, rozsiane ogniska rozrzedzenia struktury kostnej kręgów uwidocznionych w badaniu, mogące odpowiadać zmianom o charakterze przerzutowym. Obraz guza w TK klatki piersiowej przedstawiono na rycinie 2.

Dziewczynka przekazana została do Kliniki Transplantacji Szpiku, Onkologii i Hematologii Dziecięcej celem dalszej diagnostyki i leczenia. Po makroskopowo całkowitej resekcji guza, na podstawie wyniku badania histopatologicznego, zostało ustalone rozpoznanie: ganglineuroblastoma nodular wariant, stroma rich type, bez amplifikacji onkogenu MYCN. Po zabiegu dziewczynka czuła się dobrze, zgłaszane wcześniej dolegliwości bólowe brzucha ustąpiły. Po około 3 miesiącach od resekcji, dziewczynka zaczęła uskarżać się na bóle pleców zlokalizowane na pograniczu kręgosłupa piersiowego i lędźwiowego. Wykonana kilkakrotnie scyntygrafia kości (MIBG) oraz badanie MRI kręgosłupa nie potwierdziły wznowy miejscowej ani obecności ognisk przerzutowych. Obecnie dziewczynka pozostaje pod opieką tutejszej kliniki z powodu przewlekłych zaparć i nawracających objawów o charakterze refluksu żołądkowo-przełykowego bez cech zapalenia przełyku (w endoskopii prawidłowy obraz błony śluzowej górnego odcinka przewodu pokarmowego). Objęta jest również stałą opieką Kliniki Transplantacji Szpiku, Onkologii i Hematologii Dziecięcej bez cech wznowy procesu nowotworowego.

**Ryc. 1:**
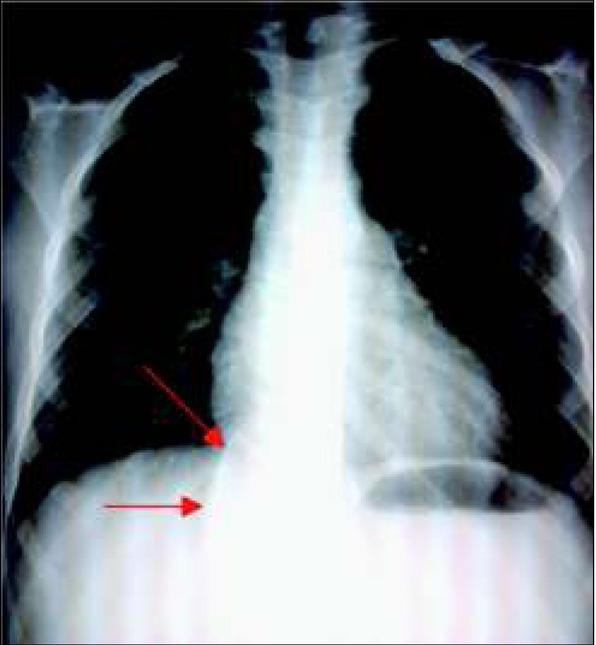
Zdjęcie RTG klatki piersiowej, w projekcji AP (strzałkami zaznaczono widoczny cień krągły) Fig. 1. A chest X-ray, anteroposterior view, showing a nodule (marked by arrows).

**Ryc. 2:**
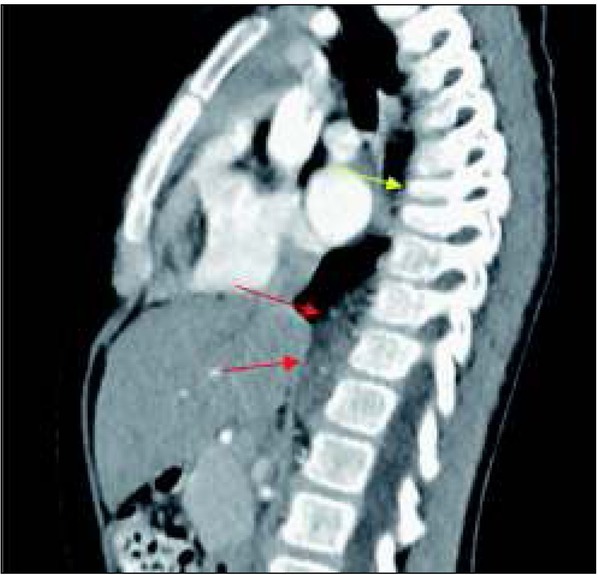
Obraz TK klatki piersiowej z kontrastem z uwidocznionym guzem okolicy przykręgosłupowej (czerwone strzałki) i złamaniem kompresyjnym trzonów kręgów (żółta strzałka). Fig. 2. Contrast computed tomography scan showing a paravertebral tumor (red arrows) and compression fractures of vertebral bodies (yellow arrow).

## Omówienie

Według klasycznej definicji Apleya i Naisha, nawracający ból brzuch charakteryzuje wystąpienie co najmniej 3 epizodów bólu brzucha w ciągu 3 miesięcy, o nasileniu ograniczającym normalną aktywność [8]. Jednak w zaburzeniach czynnościowych za przewlekły uznaje się ból brzucha utrzymujący się przez okres co najmniej 2 miesięcy w sposób ciągły lub gdy nawraca co najmniej raz w tygodniu [9]. Zaburzenia czynnościowe przewodu pokarmowego są również najczęściej przyczyną zgłaszanych dolegliwości bólowych. U niewielkiego odsetka dzieci podłożem będzie jednak schorzenie organiczne [10]. Do częstych przyczyn organicznych przewlekłych bólów brzucha u dzieci należą: zakażenia żołądkowo-jelitowe, nietolerancje i alergie pokarmowe, choroba refluksowa przełyku, przewlekle zapalenie żołądka i dwunastnicy, z lub bez zakażenia *H. pylori*, choroba trzewna oraz zakażenia w obrębie układu moczowego. Wśród rzadszych przyczyn należy uwzględnić: bezoar, zapalenie wątroby i trzustki, kamicę żółciową, kamicę układu moczowego. Natomiast do przyczyn bardzo rzadkich zaliczamy eozynofilowe zapalenie przełyku, nieswoiste zapalenia jelit, nawracające wgłobienia, uchyłek Meckela, torbiele dróg żółciowych, schorzenia ginekologiczne (torbiele jajników), chłoniaki, guzy zlokalizowane w obrębie jamy brzusznej [1, 3].

Zadaniem lekarza, a jednocześnie wyzwaniem, jest ustalenie w którym przypadku przyczyna może być organiczna i wymaga poszerzenia diagnostyki. Częstość, nasilenie, lokalizacja bólu oraz jego wpływ na ograniczenie aktywności chorego nie wykazują wartości w różnicowaniu przyczyny dolegliwości. Również często zgłaszane objawy towarzyszące w postaci niechęci do jedzenia, nudności, wymiotów, wzdęć, czy współistniejące zaburzenia o charakterze psychologicznym, nie pozwalają jednoznacznie różnicować przyczyn organicznych i czynnościowych [11]. Jednak obecność objawów alarmowych w badaniu podmiotowym i przedmiotowym oraz wywiadzie rodzinnym, u dzieci z przewlekłymi bólami brzucha sugeruje wyższe prawdopodobieństwo organicznej przyczyny dolegliwości i uzasadnia przeprowadzenie poszerzonej diagnostyki. Należy również uwzględnić możliwość współwystępowania lub nakładania się zaburzeń o charakterze czynnościowym na schorzenia organiczne [12, 13].

Objawy alarmowe w wywiadzie oraz badaniu przedmiotowym wzbudzające podejrzenie choroby organicznej przedstawiono w tabeli I [1, 3, 14].

**Tabela I:** Objawy alarmowe występujące u dzieci z przewlekłymi bólami brzucha. Table I. Alarm symptoms or signs in children with chronic abdominal pain.

Objawy alarmowe w wywiadzie u dzieci z przewlekłymi bólami brzucha *Alarm symptoms in a medical history of children with chronic abdominal pain*	
–	niezamierzony ubytek masy ciała
	*history of unwitting weight loss*
–	spowolnienie tempa wzrastania
	*history of poor growth*
–	opóźnienie dojrzewania płciowego
	*delayed puberty*
–	krwawienie z górnego lub dolnego odcinka przewodu pokarmowego (jawne lub utajone)
	*bleeding from the upper or lower gastrointestinal tract (overt or latent)*
–	długotrwałe lub podbarwione żółcią wymioty
	*long-lasting or biliary vomiting*
–	trudności lub ból w trakcie połykania (dysfagia, odynofagia)
	*difficulty or pain during swallowing (dysphagia, odynophagia)*
–	przewlekła nasilona biegunka
	*chronic, severe diarrhea*
–	ból lub biegunka w nocy
	*nocturnal pain or diarrhea*
–	nawracające, niejasne stany gorączkowe
	*recurrent unexplained fever*
–	zaburzenia w oddawaniu moczu: dysuria, hematuria, ból zlokalizowany w okolicy lędźwiowej
	*impaired urination: dysuria, hematuria, pain located in the lumbar area*
–	w wywiadzie rodzinnym: nieswoiste zapalenia jelit, choroba trzewna, choroba wrzodowa
	*inflammatory bowel disease, celiac disease, peptic ulcer disease in the family history*
–	niedokrwistość
	*anemia*
–	lokalizacja bólu z dala od pępka, szczególnie w prawym górnym lub dolnym kwadrancie brzucha
–	*localization of pain away from the umbilical region, especially in the upper or lower right abdominal quadrant*
–	wyczuwalne opory w obrębie jamy brzusznej
–	*palpable mass in the abdomen*
–	powiększenie wątroby lub śledziony
–	*hepato- or splenomegaly*
–	tkliwość lub ból w kącie żebrowo-kręgosłupowym lub w przebiegu kręgosłupa
–	*tenderness or pain in the rib-spine angle or in the course of the spine*
–	zmiany okołoodbytnicze (kłykciny, szczeliny, przetoki)
–	*perianal abnormalities (condylomas, fissures, fistulas)*
–	obecność aft w jamie ustnej
–	*presence of aphthae in the mouth*

Według obowiązujących IV Kryteriów Rzymskich do jednostek przebiegających z czynnościowym bólem brzucha należą: dyspepsja czynnościowa, zespół jelita drażliwego, migrena brzuszna oraz nieokreślony czynnościowy ból brzucha. Również zaparciom czynnościowym mogą towarzyszyć przewlekłe bóle brzucha [9]. Ból brzucha o charakterze czynnościowym nie posiada ściśle zdefiniowanych markerów diagnostycznych. Obecnie uważa się, że zaburzenia czynnościowe powinny być rozpoznawane, gdy po przeprowadzeniu odpowiedniej oceny klinicznej, objawów nie można przypisać innemu stanowi chorobowemu. Podstawą rozpoznania schorzeń czynnościowych są kryteria diagnostyczne zawarte we wspominanych IV Kryteriach Rzymskich oraz dokładnie przeprowadzony wywiad i badanie przedmiotowe. Celowość wykonania badań dodatkowych należy rozważyć w oparciu o całość obrazu chorobowego, w przypadku braku objawów alarmowych postępowanie diagnostyczne może być ograniczone do minimum. W tym kontekście rozpoznanie zaburzeń czynnościowych przestaje być rozpoznaniem „z wykluczenia”, a poszerzenie diagnostyki jest konieczne jedynie w przypadku występowania ”czerwonych flag” [9, 15].

W omawianym przez nas przypadku, u dziewczynki bóle brzucha o zmiennym nasileniu występowały z częstotliwością od kilku razy dziennie do kilku razy w miesiącu, od około 2 lat. Od okresu niemowlęcego obserwowano zaparcia, które początkowo spełniały kryteria dla rozpoznania schorzenia czynnościowego: typowy początek związany ze zmianą diety, brak objawów alarmowych, bolesne wypróżnienia i twarde stolce oddawane 2x w tygodniu, postawa retencyjna [9]. W toku prowadzonej diagnostyki rozpoznano jednak nietolerancję laktozy oraz alergię pokarmową, schorzenia organiczne, które mogą być przyczyną nawracających bólów brzucha i zaparć [1, 16]. Ze względu na obecność w wywiadzie z ostatnich kilku tygodni, objawów alarmowych w postaci bólu o stałej lokalizacji w lewym górnym kwadrancie brzucha (okolica lewego podżebrza), wybudzającego w nocy ze snu, niezamierzonego ubytku masy ciała oraz przewlekle utrzymującego się suchego kaszlu, ustalono wskazania do poszerzenia diagnostyki.

We wstępnej diagnostyce przewlekłych bólów brzucha oprócz dokładnego badania podmiotowego i przedmiotowego z uwzględnieniem objawów alarmowych, należy wykonać podstawowe badania laboratoryjne, do których należą: morfologia krwi obwodowej z rozmazem, wykładniki stanu zapalnego (OB, CRP), enzymy trzustkowe i wątrobowe, badanie przesiewowe w kierunku choroby trzewnej, badanie kału na obecność krwi utajonej i badanie biochemiczne moczu. Diagnostyka obrazowa w początkowym etapie powinna obejmować badanie ultrasonograficzne jamy brzusznej [3, 14]. Na każdym etapie diagnostyki niezmiernie ważne jest staranne ustalenie wskazań do planowanych badań. Przykładowo, u dzieci nie zgłaszających objawów alarmowych, nieprawidłowości w badaniu ultrasonograficznym brzucha znajdowane są z częstością mniejsza niż 1% [11]. W omawianym przypadku, utrzymujący się suchy kaszel i lokalizacja bólu na pograniczu klatki piersiowej i jamy brzusznej skłaniało również do wykonania zdjęcia RTG klatki piersiowej. Uwidoczniony na wysokości przepony cień krągły, biorąc również pod uwagę obecność w wywiadzie objawów refluksowych, mógł odpowiadać przepuklinie przeponowej wślizgowej. Wykluczenie w badaniu kontrastowym przepukliny przeponowej, wymagało poszerzenia diagnostyki o badanie metodą tomografii komputerowej. Uwidoczniona w badaniu TK zlokalizowana przykręgosłupowo, hipodensyjna masa z drobnymi zwapnieniami oraz obecność zmian o charakterze złamań kompresyjnych kręgów i rozsiane ogniska rozrzedzenia struktury kostnej w trzonach kręgów, przemawiała za procesem rozrostowym z o charakterze neuroblastoma.

Neuroblastoma (NB), ganglioneuroblastoma (GNB) i ganglioneuroma (GN) należą do guzów współczulnego układu nerwowego wywodzących się z prymitywnych komórek współczulnych zwojów nerwowych [6]. Stanowią one około 7% wszystkich nowotworów diagnozowanych u dzieci oraz należą do najczęściej rozpoznawanych litych guzów zewnątrzczaszkowych, z roczną zapadalnością 7,6/1,000,000 dzieci [6, 7]. Najczęściej rozpoznawane są w pierwszej dekadzie życia (80% przypadków poniżej 5 r.ż.). Neuroblastoma należy do guzów o zdecydowanie złośliwym charakterze, podczas gdy ganglineuroma zaliczana jest do guzów łagodnych. Ganglioneuroblastoma, określana jako zmiana o pośredniej złośliwości, jest guzem bardziej dojrzałym i wyżej zróżnicowanym niż neruoblastoma, o utkaniu mieszanym, w skład którego wchodzą neuroblasty i dojrzałe komórki zwojowe. Według klasyfikacji *International Neuroblastoma Pathology Classification* wyróżnia się dwa podtypy histopatologiczne: GNB intermixed (Schwannian stroma-rich) oraz GNB nodular (composite Schwannian stroma-rich/stroma-dominant and stroma-poor), o zdecydowanie lepszym rokowaniu w przypadku GNB intermixed [6, 17]. Ganglioneuroblastoma rozpoznawana jest z jednakową częstością u obu płci, najczęściej przed 10 r.ż. (średni wiek 7 r.ż.) [18]. Może występować w różnej lokalizacji, wszędzie tam gdzie znajdują się komórki układu współczulnego. Najczęściej guz zlokalizowany jest w obrębie nadnerczy (35% przypadków), zwojach międzykręgowych przestrzeni zaotrzewnowej (30-35%) oraz śródpiersiu tylnym (20%). W niewielkim odsetku przypadków znajdowany jest w obrębie głowy, szyi, miednicy i płuc [19]. Obraz kliniczny choroby jest bardzo zróżnicowany i w dużej mierze determinowany lokalizacją zmiany. Guzy położone w obrębie jamie brzusznej mogą powodować bóle brzucha, wymioty, powiększenie obwodu brzucha, biegunki oraz objawy związane z wnikaniem do kanału kręgowego i uciskiem rdzenia: zaburzenia mikcji, zaburzenia oddawania stolca (zaparcia), przymusowe ułożenie ciała, przeczulicę. Lokalizacja zmiany w środkowej lub dolnej części klatki piersiowej najczęściej przebiega bezobjawowo, może jednak powodować duszność, kaszel, stridor, ból w klatce piersiowej, zaburzenia połykania oraz bóle pleców. W przebiegu choroby występują czasem objawy ogólne w postaci utraty masy ciała, podwyższonej temperatury, senności, bladości powłok, osłabienia, drażliwości, niepokoju, czasem również obwodowe objawy neurologiczne. Z uwagi na złośliwy charakter guza, czasami obserwuje się przerzuty do szpiku kostnego, kości, wątroby, płuc, rzadko do skóry (sinawe przebarwienia skóry), co również może wpływać na obraz kliniczny choroby. W prawie połowie przypadków guz wykrywany jest przypadkowo w badaniu RTG klatki piersiowej lub USG jamy brzusznej [6, 19]. Podobnie w przypadku diagnozowanej przez nas dziewczynki, guz został wykryty niejako przypadkowo w badaniu radiologicznym klatki piersiowej, jednak prezentowane wcześniej objawy (zlokalizowany kłujący ból, dolegliwości nocne, zaburzenia defekacji, przewlekły kaszel, ubytek masy ciała), chociaż dość skąpe, mogły wskazywać na proces rozrostowy w obrębie jamy brzusznej.

Rozpoznanie guzów neuroblastycznych opiera się przede wszystkim na badaniach obrazowych. W badaniu ultrasonograficznym guz może być opisany jako niejednorodna echogenicznie masa ze zwapnieniami i cechami rozpadu. Badaniem z wyboru pozostaje tomografia komputerowa, która umożliwia dokładniejszą ocenę pochodzenia, rozmiarów i lokalizacji guza, występowania zwapnień, zajęcia regionalnych węzłów chłonnych, stopnia naciekania sąsiadujących tkanek. Rezonans magnetyczny (MRI) jest wykorzystywany do oceny naciekania pierwotnego guza na struktury kanału kręgowego oraz do wykrywania przerzutów do wątroby u niemowląt i małych dzieci [19]. Guzy pochodzące z komórek układu współczulnego produkują katecholaminy, co wykorzystywane jest jako marker diagnostyczny. Podwyższony poziom katecholamin w osoczu lub ich metabolitów w dobowej zbiórce moczu (kwas wanilinomigdałowy i homowanilinowy) przemawia za rozpoznaniem choroby. Ostateczne rozpoznanie stawiane jest na podstawie histopatologicznego potwierdzenia choroby w bioptacie guza lub biopsji szpiku. Obligatoryjne w celu oceny stopnia zaawansowania choroby wykonywane są: biopsja szpiku oraz scyntygrafia kości z użyciem znakowanej jodem 123-metajodobenzylguanidyny (MIBG) [19]. Potwierdzenie w badaniu cytogenetycznym zwiększonej liczby kopii onkogenu MYCN stanowi niekorzystny czynnik rokowniczy. Wysokie wartości stężenia ferrytyny, dehydrogenazy mlecznowej (LDH) oraz neuronospecyficznej enolazy (NSE) w surowicy są również złym czynnikiem prognostycznym [20]. Leczenie uzależnione jest od stopnia zaawansowania choroby określonego według klasyfikacji *International Neuroblastoma Risk Group Staging System* (INRGSS) [21]. W przypadku zlokalizowanej choroby bez czynników ryzyka wystarczające jest chirurgiczne usunięcie zmiany. W stopniach bardziej zaawansowanych stosuje się skojarzone leczenie chirurgiczne, chemio- i radioterapię oraz autogeniczny przeszczep komórek macierzystych szpiku [22].

W przypadku ganglioneuroblastoma rokowanie co do odpowiedzi na leczenie i długości przeżycia jest zdecydowanie lepsze niż dla neuroblastoma. Guz może ulegać spontanicznej regresji (1-2%) lub różnicowaniu do ganglioneuroma. Łagodniejszy przebieg kliniczny i lepsze rokowanie obserwowane jest również w przypadku pacjentów z GNB intermixed w porównaniu do podtypu nodular [6, 21].

## Podsumowanie

Dokładny wywiad i badanie przedmiotowe wraz z uwzględnieniem objawów alarmowych oraz odpowiednio dobrane badania dodatkowe, stanowią kluczowe narzędzia w identyfikacji pacjentów z chorobą organiczną. W diagnostyce różnicowej należy również uwzględnić rzadkie przyczyny przewlekłych bólów brzucha, w tym możliwość wystąpienia choroby onkologicznej. Obraz kliniczny guzów neuroblastyczych jest niezwykle różnorodny, a lekarze powinni być świadomi nie tylko klasycznych objawów choroby, ale również tych nietypowych. Ustalenie wskazań do poszerzenia diagnostyki pozwala na szybkie postawienie rozpoznania i wdrożenie odpowiedniego postępowania terapeutycznego, co przekłada się na poprawę wskaźników przeżycia i zminimalizowanie nieodwracalnych uszkodzeń.
